# Phytochemical composition of *Potentilla anserina* L. analyzed by an integrative GC-MS and LC-MS metabolomics platform

**DOI:** 10.1007/s11306-012-0473-x

**Published:** 2012-11-17

**Authors:** Angela Mari, David Lyon, Lena Fragner, Paola Montoro, Sonia Piacente, Stefanie Wienkoop, Volker Egelhofer, Wolfram Weckwerth

**Affiliations:** 1 Department of Pharmaceutical and Biomedical Sciences, University of Salerno, Salerno, Italy; 2 Department of Molecular Systems Biology, University of Vienna, Vienna, Austria

**Keywords:** Medicinal plants, Metabolomics, GC-MS, LC-MS, Flavonoids, Genistein, Mass accuracy precursor alignment (MAPA)

## Abstract

*Potentilla anserina* L. (Rosaceae) is known for its beneficial effects of prevention of pre-menstrual syndrome (PMS). For this reason *P. anserina* is processed into many food supplements and pharmaceutical preparations. Here we analyzed hydroalcoholic reference extracts and compared them with various extracts of different pharmacies using an integrative metabolomics platform comprising GC-MS and LC-MS analysis and software toolboxes for data alignment (MetMAX Beta 1.0) and multivariate statistical analysis (COVAIN 1.0). Multivariate statistics of the integrated GC-MS and LC-MS data showed strong differences between the different plant extract formulations. Different groups of compounds such as chlorogenic acid, kaempferol 3-*O*-rutinoside, acacetin 7-*O*-rutinoside, and genistein were reported for the first time in this species. The typical fragmentation pathway of the isoflavone genistein confirmed the identification of this active compound that was present with different abundances in all the extracts analyzed. As a result we have revealed that different extraction procedures from different vendors produce different chemical compositions, e.g. different genistein concentrations. Consequently, the treatment may have different effects. The integrative metabolomics platform provides the highest resolution of the phytochemical composition and a mean to define subtle differences in plant extract formulations.

**Electronic supplementary material:**

The online version of this article (doi:10.1007/s11306-012-0473-x) contains supplementary material, which is available to authorized users.

## Introduction

*Potentilla anserina* L. (silverweed) belongs to the family of Rosaceae and its extracts have been used for a long time in traditional medicine. The gynecological indication for *P. anserina* is based on pharmacological studies showing that the herb increases the tonus of the isolated uterus in various animal species (Schulz et al. 1998). Additionally, extracts of the aerial and/or underground parts have been applied in traditional medicine for the treatment of inflammations, wounds, certain forms of cancer, infections due to bacteria, fungi and viruses, diarrhoea, diabetes mellitus and other ailments (Bundesgesundheitsamt 1985, 1990). Tomczyk and Latté report that *P. anserina* (aerial parts or the whole plant) and other *Potentilla* species are generally used to prepare homeopathic medications (Tomczyk and Latté 2009) according to homeopathic pharmacopoeias like Homeopathic Pharmacopoeia of the United States (HPUS) and German Homeopathic Pharmacopoeia (HAB) (Hiller 1994). For this reason *P. anserina* is processed into many food supplements and pharmaceutical preparations such as teas, tinctures, capsules, tablets, and juice and is consumed by women in order to prevent the symptoms of pre-menstrual syndrome (PMS).

**Fig. 1:**
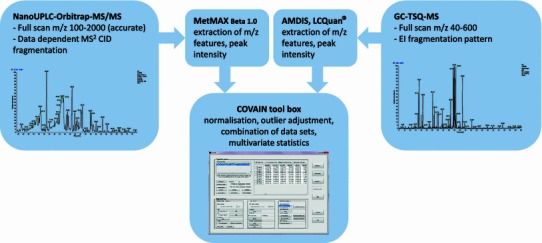
Metabolomics platform for the characterization of medicinal plants integrating GC-MS, LC-MS, alignment tools and statistical analysis using COVAIN (Sun and Weckwerth 2012)

Despite all these positive effects, so far only limited analytical information of the chemical composition on *P. anserina* is available (Swiezewska and Chojnacki 1989; Kombal and Glasl 1995; Schimmer and Lindenbaum 1995; Tomczyk et al. 2010; Xu et al. 2010). In particular mass spectrometric data of the chemical composition of *P. anserina* are still lacking. These could however be helpful for the evaluation of physiological properties of individual plant secondary metabolites and for stability studies of pharmaceutical preparations. HPLC coupled to mass spectrometry (LC-MS) proved to be a very useful tool and is largely applied to the characterization of plant secondary metabolites. Gas chromatography coupled to mass spectrometry (GC-MS) provides complementary data to LC-MS analysis comprising small polar chemicals such as organic acids, sugars, amino acids, sugar alcohols and many more (Scherling et al. 2010; Weckwerth 2010). The aim of the present work is to characterize the phytochemical profile of hydroalcoholic extracts of *P. anserina* and its commercial products prepared by different pharmacies using a comprehensive metabolomics platform integrating GC-MS, LC-MS and multivariate statistics (Fig. 1).

## Materials and methods

### Chemicals

Chloroform, ethanol (absolute), methanol, *n*-butanol, petroleum ether (HPLC grade), were purchased from Sigma-Aldrich (Vienna, Austria) in Chromasolv^®^ grade as well as pyridine over molecular sieve (GC-grade). Acetonitrile (HPLC grade) and formic acid were obtained from Merck (Darmstadt, Germany). Chlorogenic acid was purchased from Roth (Graz, Austria), genistein, quercetin-3-*O*-glucoside and D-sorbitol, β-alanine, arabinose, asparagine, citric acid, d(-)quinic acid, fructose, glucose, glyceric acid, glycerol, glycine, l-alanine, l-aspartic acid, l-leucine, l-serine, l-threonine, malic acid, mannitol, myo-inositol, phenylalanine, pinitol, proline, sucrose, trehalose, valine and, xylose were purchased from Sigma-Aldrich (Vienna, Austria), kaempferol-3-*O*-glucoside was obtained from Extrasynthese (Genay, France). Myricetin-3-*O*-rhamnoside and quercetin-3-*O*-glucuronide were isolated by size exclusion chromatography and semipreparative HPLC with UV detection from the hydroalcoholic extract of *P. anserina* and their structures were characterized by 1D and 2D nuclear magnetic resonance (NMR). For derivatization methoxyamine hydrochloride and *N*-methyl-*N*-trimethylsilyltrifluoroacetamide (MSTFA) were purchased from Sigma-Aldrich (Vienna, Austria), as well as retention index marker alkanes (all even C_10_–C_40_). HPLC grade water (18.2 mΩ) wËas prepared using a Millipore Milli-Q purification system (Millipore Corp., Bedford, MA, USA).

### Plant material

*Potenilla anserina* air-dried plant parts were purchased from Minardi s.r.l. (Bagnacavallo, Ra, Italy). 500 g of whole plant parts were extracted with petroleum ether three times. After filtration the raw material was extracted three times with chloroform and finally with 70 % EtOH following the same procedure performed with petroleum ether. The collected alcohol-aqueous extract (PanserinaUniSa) was dried under vacuum.

A second extract (PanserinaUniVie) was prepared using a grinding mill system MM400 from Retsch (Haan, Germany). 250 mg of ground plant material was extracted with 25 mL of a solution of methanol/chloroform/water (2.5:1:0.5, v:v:v) (Weckwerth et al. 2004) and then vortexed for 10 min followed by 8 min incubation. The sample was then centrifuged for 4 min at 3,400×*g* and the supernatant was separated from the pellet. 5 mL of distilled water were added to the supernatant, followed by 10 s shaking on a vortex and 2 min centrifugation at 3,400×*g*. The alcoholic-aqueous phase was dried under vacuum.

Five mother tinctures were acquired from five drugstores (# 1, 2, 3, 4 and 5) in Vienna. For each of them 1 mL was dried under vacuum.

All samples were analyzed by gas chromatography and liquid chromatography coupled to mass spectrometry. For data analysis (see below) all sample injections were normalized against corresponding extract dry weights.

### Extract derivatization and GC-MS analysis

The protocol for GC-MS analysis was performed according to Weckwerth et al. (2004) with slight changes. Before derivatization 25 μL of ^13^C-d-sorbitol (0.02 μg μL^−1^) were added to all samples as internal standard. Samples were derivatized in two steps. First 20 μL methoxyamination mixture (40 mg mL^−1^ methoxyamine hydrochloride in dry pyridine) were added and incubated for 90 min at 30 °C in a thermo shaker. Then 80 μL of *N*-methyl-*N*-trimethylsilyltrifluoroacetamide (MSTFA) silylation mixture including retention index marker were added (30 μL of alkane mixture (even-numbered C10-C40-alkanes, each 50 mg L^−1^) and incubated for 30 min at 37 °C.

Derivatized samples were centrifuged and 50 μL of supernatant was transferred to GC-vials with micro inserts and closed with crimp caps.

GC-MS analyses were performed on a ThermoFisher Trace gas chromatograph coupled to a Triple Quadrupole mass analyzer (Thermo Scientific TSQ Quantum GC™, Bremen, Germany). 1 μL of derivatized sample was injected at a constant temperature of 230 °C in splitless mode with a deactivated Siltek liner (Restek). Each sample was measured three times with the same conditions to get technical replicates.

GC separation was performed on a HP-5MS capillary column (30 m × 0.25 mm × 0.25 μm) (Agilent Technologies, Santa Clara, CA), at a constant flow 1 mL min^−1^ helium. Initial oven temperature was set to 70 °C and hold for 1 min, followed by a ramp to 76 °C at 1 °C min^−1^ and a second ramp at 6 °C min^−1^ to 350 °C hold for 1 min. Transfer line temperature was set to 340 °C and post run temperature to 325 °C for 10 min.

Mass analyzer was used in full scan mode scanning a range from *m/z* 40–800 at a scan time of 250 ms. Electron impact (EI) ionization was used at 70 eV and ion source temperature was set to 250 °C.

Metabolite derivatives were identified by matching retention time as well as mass spectra (see Table 1) with those of the corresponding reference standards and by comparison with an in house mass spectral library. Metabolites were considered identified with a spectral match factor higher than 850 and RI-deviation lower than 10. Deconvolution was performed with AMDIS (Stein 1999) and quantification with LC-Quan2.6.0 (Thermo Fisher Scientific Inc.). For statistical analyses a Matlab tool called COVAIN was used that provides a complete workflow including uploading data, data preprocessing, data integration and uni- and multivariate statistical analysis (Sun and Weckwerth 2012).

**Table 1:** Compounds occurring in *P. anserina* hydroalcoholic extracts measured by GC/EI(TSQ)MS

Compounds	Rt (min)	Fragments *m/z*
1. Glycolic acid (2TMS)	9.50	147
2. Alanine (2TMS)^a^	10.28	116
3. Unknown 1	11.36	133
4. Unknown 2	12.59	281
5. Valine (2TMS)^a^	13.53	144
6. Leucine (2TMS)^a^	15.03	158
7. Glycerol (3TMS)^a^	15.24	205
8. Proline (2TMS)^a^	15.59	142
9. Glycine (3TMS)^a^	15.81	174
10. Succinic acid (2TMS)	16.06	247
11. Glyceric acid (3TMS)^a^	16.60	189
12. Serine (3TMS)^a^	17.30	204
13. Threonine (3TMS)^a^	17.91	218
14. Malic acid (3TMS)^a^	20.18	233
15. Pyroglutamic acid (2TMS)	20.57	156
16. Threitol or erythritol (4TMS)	20.67	156
17. Aspartic acid (3TMS)^a^	20.73	232
18. 4-amino butyric acid (3TMS)	20.79	174
19. Unknown 3	21.73	292
20. Unknown 4	22.13	307
21. Phenylalanine (2TMS)^a^	22.67	192
22. Asparagine (3TMS)^a^	23.61	188
23. Arabinose (1MeOx) (4TMS)^a^	23.76	103
24. Xylose (1MeOx) (4TMS)^a^	24.05	217
25. Xylitol or ribitol (5TMS)	24.80	217
26. 2-desoxy-pentos-3ylose (2MeOx)(2TMS)	25.30	231
27. Unknown 5	25.79	257
28. Lyxonic acid (5TMS)	25.90	292
29. Shikimic acid (4TMS)	26.25	204
30. Carbohydrate	26.30	217
31. Citric acid (4TMS)^a^	26.43	273
32. Carbohydrate (5TMS)	26.50	204
33. Glucopyranoside (5TMS)	26.64	204
34. Pinitol (5TMS)^a^	26.76	217
35. Quinic acid (5TMS)^a^	27.24	345
36. Fructose (1MeOx) (5TMS)^a^	27.60	307
37. Hexose (5TMS)	27.78	191
38. Glucose (1MeOx) (5TMS)^a^	27.91	319
39. Mannitol (6TMS)^a^	28.43	319
40. Unknown (inositol isomer)	28.82	305
41. Glucopyranoside (5TMS)	29.27	217
42. Carbohydrate (glucopyranoside 5TMS)	29.28	204
43. Gluconic acid (6TMS)	29.59	333
44. Unknown 6	30.04	204
45. *Myo*-inositol^a^ (6TMS)	30.89	305
46. Sucrose (8TMS)^a^	38.61	361
47. Trehalose (8TMS)^a^	39.83	361
48. Isomaltose (1MeOx) (8TMS)	40.89	361
49. Melibiose (1MeOx) (8TMS)	41.16	204
50. Unknown 7	41.77	369
51. Unknown 8	44.22	204
52. Unknown 9	44.35	647
53. Unknown 10	45.38	575

Given are (methoxime)-trimethylsilyl [(MeOx) (TMS)] derivatives of metabolites including their retention time (Rt) and EI-fragments taken for relative quantification

^a^Confirmed by comparison with corresponding reference standard

### NanoLC-Orbitrap-MS/MS analyses

For all the samples described in the plant material section, 0.12 μg μL^−1^ water/acetonitrile (95:5, v:v) 0.1 % formic acid solutions were prepared and centrifuged at 13,000×*g* for 3 min. For each of them, 5 μL were used for LC-MS and MS/MS analysis in triplicates.

A 1D plus nanoUHPLC system (Eksigent, Dublin, Ireland) was equipped with an autosampler and the employed column was a Waters nanoAcquityHSS T_3_, 1.8  μm, 100 μm  ×  100 mm. The mobile phases were water 0.1 % formic acid (A) and 90 % acetonitrile in water 0.1 % formic acid (B) at a flow rate of 500 μL min^−1^. The LC conditions were 5 % B during 0–3 min, a linear increase from 5 to 20 % B during 3–25  min, from 20 to 40 % B during 25–40  min and from 40 to 50 % B during 40–55 min, finally from 50 to 95 % B during 55–63 min followed by 15 min of maintenance. A Thermo Electron LTQ-Orbitrap XL mass spectrometer equipped with a nano electrospray ion source (ThermoFisher Scientific, Bremen, Germany) and operated under Xcalibur 2.1 version software, was used in positive ionization mode for the MS analysis using data-dependent automatic switching between MS and MS/MS acquisition modes. The instrument was calibrated using the manufacturer’s calibration standards. The scan was collected in the Orbitrap at a resolution of  30, 000 in a *m*/*z* range of 150–1,800. In order to achieve even higher mass accuracy a lock mass option was enabled in both MS and MS/MS mode and the cyclomethicone N5 ions generated in the electrospray process from ambient air (*m*/*z* = 371.101230) were used for internal recalibration in real time. This allowed mass accuracies of <1 ppm. The capillary voltage was 4.5  kV, the tube lens offset 160 V and the capillary temperature was set at 180 °C, no sheath gas and auxiliary gas were used.

Data deconvolution was performed with a modified ProtMAX version called MetMAX Beta 1.0 which provides mass accuracy precursor alignment of selected *m/z* signals in the LC-MS profile (Hoehenwarter et al. 2008). As for GC-MS, the COVAIN tool (Sun and Weckwerth 2012) was used for statistical analyses of the LC-MS data as well.

### MetMAX Beta 1.0 processing and COVAIN analysis of LC-MS data

Raw data files were converted to mzXml format using the MassMatrix mass spectrometric data file conversion tool version 3.9 from the Case Western Reserve University (Cleveland, Ohio, USA; http://www.massmatrix.net/). MetMAX Beta 1.0 was used to process the mzXml files, generating a matrix of precursor ion intensities (Hoehenwarter et al. 2008). Each column vector contains the quantities of selected metabolites; each row vector describes the abundance of a respective metabolite ion over the entire set of analyses. Each column was normalized to its total spectral count. The .csv data table resulting from MetMAX Beta 1.0 were imported into COVAIN for statistical analysis (Sun and Weckwerth 2012). The values were then log-transformed. Principal component analysis (PCA) was performed for decomposition and visualization of data. The components of the column vectors, i.e. the precursor *m/z*, constitute the loadings of the independent components, and were identified by matching their retention times and mass spectra with those of the corresponding reference standards (see supplementary data).

## Results and discussion

### Qualitative and quantitative nanoLC-Orbitrap-MS/MS analyses of *P.anserina* crude extract

**Fig. 2:**
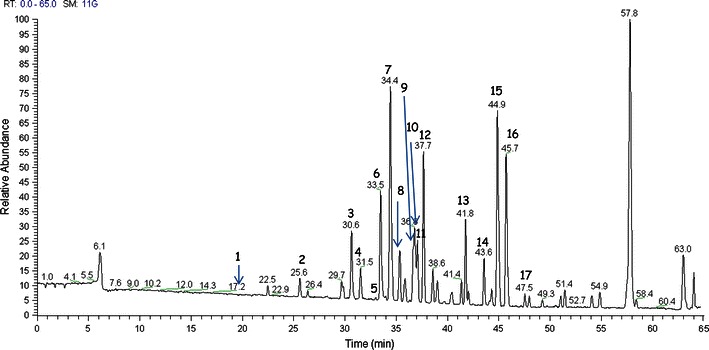
NanoLC-Orbitrap-MS profile (full MS-mode) of the crude hydroalcoholic extract of *P. anserina* (positive ion mode) (see also Table 2)

In order to obtain a metabolite profile of the crude extract of *P. anserina*, an analytical method based on nanoLC-Orbitrap-MS/MS was developed. The LC-MS profile highlighted the presence of a large group of compounds corresponding to the protonated molecular ions of different flavonoids and caffeoylquinic acids (Fig. 2).

Individual components were identified by comparison of their *m/z* values in the Total Ion Count (TIC) profile with those of the selected compounds described in literature (Table 2) or by matching their MS/MS spectra with those reported in a public repository of mass spectral data called *Mass Bank* (Horai et al. 2010). According to our knowledge compounds 1, 2, 13, 14, 15, 16, 17 were never reported in this species. The positive HR-ESI-MS spectrum of compound 1 showed a [M + Na]^+^ ion peak at *m/z* 377.0477 along with a less intense signal at *m/z* 355 corresponding to the protonated ion. The analysis of the MS/MS spectrum of the sodium adduct of compound 1, highlighted the presence of product [(M-192) + H]^+^ at *m/z* 163 a.m.u. due to the loss of a quinic acid unit. By comparing the Rt, the mass and the MS/MS spectra of compound 1 with that of the commercial reference standard we unambiguously confirmed chlorogenic acid in *P. anserina* extracts (Table 2; Fig. 2).

**Table 2:** Retention time (Rt), precursor ions and product ions (for qualitative confirmation of the compound), of compounds occurring in *P. anserina* hydroalcoholic extracts by nanoLC-Orbitrap-MS/MS

Compound	Rt (min)	Precursor ion (*m/z*)	Product ions (*m/z*)	References
1. Chlorogenic acid^b^	19.6	377.0477	355; 163	–
2. *Chlorogenic acid isomer*	25.6	377.0477	355; 163	–
3. Myricetin 3-*O*-glucuronide^a^	30.6	495.0769	319	Kombal and Glasl (1995)
4. Quercetin -3-*O*-sambubioside^a^	31.5	597.1451	465; 303	Kombal and Glasl (1995)
5. Myricetin 3-*O*-rhamnoside^b^	33.5	465.1025	319	Kombal and Glasl (1995)
6. Quercetin 3-*O*-glucoside^b^	34.0	465.1027	303	Kim et al. (2004)
7. Quercetin 3-*O*-glucuronide^b^	34.4	479.0817	303	Merfort and Wendisch (1988)
8. Quercetin 3-*O*-xyloside^a^	35.3	435.0920	303	Zou et al. (2002)
9. Quercetin pentoside	36.6	435.0921	303	–
10. Kaempferol 3-*O*-glucoside^b^	36.8	449.1077	287	Kim et al. (2004)
11. Rutin (mass bank match)	37.1	611.1815	465; 303	Wang et al. (1999)
12. Isorhamnetin3-*O*-glucuronide^a^	37.7	493.0975	317	Kombal and Glasl (1995)
13. Acacetin-7-*O*-rutinoside (mass bank match)	41.7	593.1863	447; 285	–
14. Kaempferol 3-*O*-rutinoside (mass bank match)	43.6	595.1445	449; 287	–
15. *Unknown*	45.0	668.4370	489; 471; 453;435; 409	–
16. *Unknown*	45.7	668.4371	489; 471; 453;407; 316	–
17. Genistein^b^	47.0	271.0601	253; 243; 225;215; 197; 159; 153; 145	–

In *italised names* are putative identifications of compounds, without any comparison with the corresponding reference standard or with *Mass Bank*

^a^Compounds, without any comparison with the corresponding reference standard or with *Mass Bank* but already reported in *P. anserina*

^b^Confirmed by comparison with corresponding reference standard

The analysis of the HR-ESI-MS spectrum of compound 2 suggested it as a chlorogenic acid isomer showing the diagnostic [M + Na]^+^ ion at *m/z* 377.0477 along with the [M + H]^+^ ion at *m/z* 355 with a shift of 6 min in Rt. In particular, by the analysis of the tandem mass spectrum of the [M + H]^+^ ion, compound 2 showed product ions accounting for the same composition of chlorogenic acid, originated by the neutral loss of 192 a.m.u. (Table 2; Fig. 2).

Full positive HR-ESI-MS profile of compound 13 was in agreement with a di-glycosylated acacetin structure, showing the diagnostic [M + H]^+^ ion at *m/z* 593.1864. The analysis of the ESI-MS^2^ spectrum of 13 allowed to determine the presence of a deoxy-hexose unit, besides product ion originated by the sequential neutral losses of 146 a.m.u leading to [(M-146) + H]^+^ion at *m/z* 447 and of 162 a.m.u. leading to [(M-146-162) + H]^+^ ion at *m/z* 285 corresponding to the acacetin-aglycone (Table 2; Fig. 2). For identification, the MS^2^ spectrum of compound 13 was compared to that of acacetin 7-*O*-rutinoside present in *Mass Bank* library.

The HR-ESI-MS spectrum of compound 14 assigned it to a diglycosylated kaempferol according to the presence of the [M + H]^+^ ion at *m/z* 595.1445. The tandem mass experiment on the [M + H]^+^ ion allowed to observe a product ion at *m/z* 449, due to the neutral loss of one deoxy-hexose 146 a.m.u. and a product ion at *m/*z 287, due to the neutral loss of one hexose unit and corresponding to a kaempferol-aglycon (Table 2; Fig. 2). Identification of compound 14 as kaempferol 3-*O*-rutinoside was done by matching its tandem mass spectra with that of *Mass Bank* (data not shown).

According to the HPLC-ESIMS data, the positive ESIMS spectrum of compound 17 showed a minor [M + H]^+^ ion peak at *m/z* 271.0601. Interestingly, the MS/MS spectrum of the [M + H]^+^ ion showed a fragmentation pattern very similar to what was proposed by Lee et al. (2002) for the isoflavone genistein. By comparing compound 17 Rt and MS/MS spectra to that of the corresponding commercial standard we confirmed it as genistein (Table 2; Fig. 2). Although the presence of genistein and its glycosides is already reported in the family of Rosaceae (Jung et al. 2002; Lee et al. 2002; Ismail and Hayes 2005; Tohno et al. 2010) and in *Potentilla* genus (Şöhretoğlu and Sterner 2011), this is the first time that this isoflavone is reported in this particular species.

For compounds 15 and 16 no unambiguous identification was possible. These compounds could be isorhamnetin derivatives with two glucuronide units according to their fragmentation pattern in MS/MS. The structural elucidation is planned in future studies.

### Multivariate statistical analysis of *Potentilla anserina* crude and commercial extracts from different pharmacies

In order to carry out a comparative study between our *P. anserina* reference extract and five hydroalcoholic extracts from different pharmacy vendors all were analyzed with the same GC- and LC-MS conditions.

In both cases, our results revealed that qualitative profiles of mother tinctures seem to be very similar to that of the crude extract shown in supplementary Fig. 1 and 2.

**Fig. 3:**
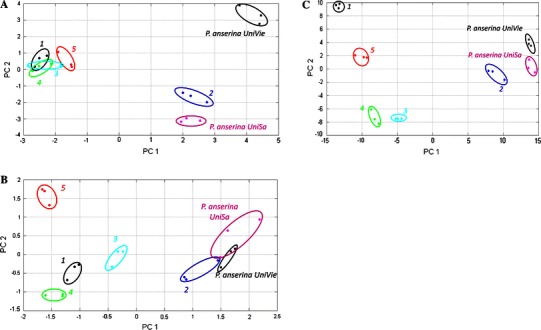
**a** Sample patterns of the hydroalcoholic extracts of *P. anserina* analyzed by GC-MS. PC1 occupies 42 % and PC2 23 % of total variance. **b** Scores plot of the hydroalcoholic extracts of *P. anserina* analyzed by LC-MS (only identified compounds used as variables). PC1 occupies 39 % and PC2 19 % of total variance. **c** Sample patterns of the hydroalcoholic extracts of *P. anserina* analyzed by LC-MS (all the compounds with RSD <25 used as variables). PC1 occupies 52 % and PC2 27 % of total variance

To better highlight the differences in metabolite profiling of the different extracts of *P. anserina*, unsupervised PCA was performed using COVAIN (Sun and Weckwerth 2012). Pre-processed GC-MS data sets (see “Materials and methods”) from the different samples were analyzed. The PCA scores plot, shown in Fig.  3a, could be readily divided into two different groups indicating that the content and distribution of components were different between the *P. anserina* crude extracts (PanserinaUniSA and PanserinaUnivie) and the respective commercial products. The corresponding PCA loadings were utilized to identify the differential metabolic compositions accountable for the separation among groups (supplementary Fig. 3 and 4 and supplementary Table 1). In the loadings plot, the Rt and *m/z* values which point far away from zero represent characteristic markers with most confidence to each group. Unknown 1 (Rt 11.36  min, *m/z* 133), proline (2TMS) (Rt 15.59  min, *m/z* 142), 2-desoxy-pentos-3ylose-dimethoxyamine (2TMS) (Rt 25.30  min, *m/z* 231), carbohydrate (Rt 26.30  min, *m/z* 217), glucopyranoside (5TMS) (Rt 26.64  min, *m/z* 204) and unknown 7 (Rt 41.77 min, *m/z* 369) (Table 1) accounted primarily for the differences among our samples.

**Fig. 4:**
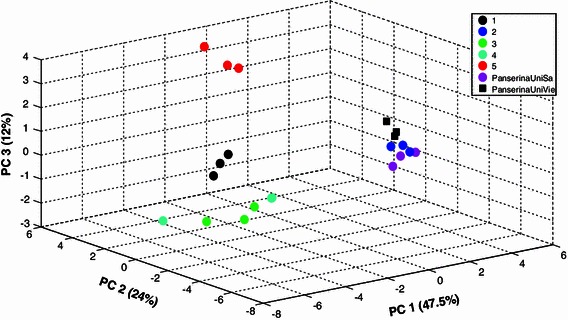
Sample patterns of combined GC-MS and LC-MS data of the hydroalcoholic extracts of *P. anserina*

The nanoLC-Orbitrap-MS/MS data of all determined samples were processed and aligned with MetMAX Beta 1.0 software by selecting a target list containing all the 17 identified ions (Table 2). The resulting data matrix containing normalized intensities of the selected peaks was further exported into COVAIN for PCA (Fig. 3b). In this latter case chlorogenic acid (Rt 19.6  min, *m/z* 377.0477), myricetin-3-*O*-glucuronide (Rt 30.6  min, *m/z* 495.0769), acacetin-7-*O*-rutinoside (Rt 41.7  min, *m/z* 593.1863) and genistein (Rt 47.0 min, *m/z* 271.0601) (Table 2) are responsible for the differences among our samples. Both GC and LC-MS PCA plots showed the same tendencies between crude extracts and commercial samples (see Figs. 3, 4). In particular it is observed that “PanserinaUniSa” and “PanserinaUnivie” can be considered to be very similar and also very close to hydroalcoholic extract “#2”. As shown in Fig. 3, extracts # 1, 3, 4 and 5 are far away from the other three samples.

To obtain a more comprehensive view of the LC-MS data a second PCA was applied to a dataset pre-processed by MetMAX Beta 1.0. After calculating the intensity mean, standard deviation and the relative standard deviation (RSD) among three technical replicates for all the peaks, the RSD mean was estimated for each peak and only those with a value <25 were selected for multivariate statistical analysis. By application of this method we selected 1,866 variables for PCA (Fig. 3c). Both plots, Fig. 3b and c, are very similar indicating the robustness of the LC-MS MetMAX Beta 1.0 approach.

Eventually the integration of GC-MS and LC-MS data into one data matrix for PCA showed the clear separation of the hydroalcoholic extracts PanserinaUniSa, PanserinaUniVie and #2 from the other extracts (Fig. 4). The loadings (supplementary Table 1) of this PCA plot demonstrates the different importance of either GC-MS or LC-MS compounds for sample classification. Synergistic effects of data integration for sample pattern recognition were also recently revealed in studies for the integration of primary and secondary metabolism as well as due to the integration of metabolomic and proteomic data (Morgenthal et al. 2005; Wienkoop et al. 2008; Doerfler et al. 2012). The integration of GC-MS and LC-MS data enables the search of precursor-product correlations in biosynthetic pathways. This was recently shown in the study by Doerfler et al. (2012) using Granger causality analysis to reveal the biosynthetic interface of primary and secondary metabolism.

### Comparative analysis of genistein in extracts from different pharmacy vendors

**Fig. 5:**
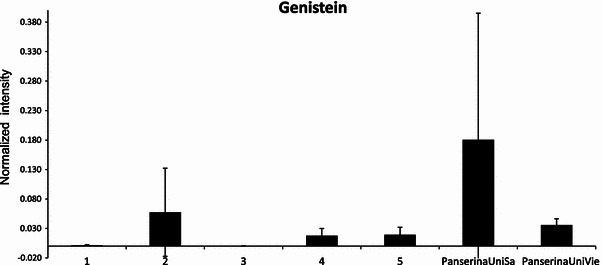
Relative quantitative analysis of genistein in the commercial products (# 1, 2, 3, 4, 5) and in the hydroaloholic extracts PanserinaUnisa and UniVie. Values are mean of triplicates for each sample. *Error bars* indicate the standard deviation (SD±) values for each histogram

Figure 5 shows the relative evaluation of genistein in all samples. The values were obtained after normalizing the LC-MS intensities of this compound against the total counts of all variables within a sample (calculated with MetMAX Beta 1.0). The results show the higher amount of this isoflavone in the extracts of PanserinaUniSa and PanserinaUniVie as well as in the commercial product 2, thus confirming the similarities between these three samples already deduced from PCA analysis. Since genistein is considered as an active compound in estrogenic therapy (Ferrante et al. 2004; Hellstrom and Muntzing 2012), our results highlight that genistein intake may change depending on the origin of different commercial products, thereby having different effects on the treatment of PMS.

## Conclusion

In this study we report for the first time a high resolution LC-MS method for the evaluation of the chemical composition of *P. anserina* polar extracts. By this accurate and sensitive analysis we revealed the presence of compounds never reported for *P. anserina*. Especially important is the identification of the isoflavone genistein which is considered as an active compound in the estrogenic therapy. This fact may explain the positive effect of *P. anserina* polar extracts in the treatment of premenstrual syndrome diseases.

Moreover our results showed the advantages of applying an integrated LC-MS, GC-MS metabolomics platform for the evaluation of the similarities between medicinal plant extracts and their commercial products. The unbiased assignment of *m*/*z* features to sample classification using Mass Accuracy Precursor Alignment (MAPA) and the corresponding MetMAX algorithm in combination with multivariate statistics [MAPA and COVAIN; (Hoehenwarter et al. 2008; Sun and Weckwerth 2012)] opens up opportunities to identify novel compounds in the medicinal plant extracts which were previously not detected. We have discussed two of these unknowns and will address these investigations in more detail in future studies.

## Electronic supplementary material

Below is the link to the electronic supplementary material.

Supplementary material 1 (XLS 55 kb)

Supplementary material 2 (PPTX 1066 kb)
