# Supplementary material for: Phytochemical composition of Potentilla anserina L. analyzed by an integrative GC-MS and LC-MS metabolomics platform
Source: Metabolomics. 2012 Nov 17;9(3):599–607. doi: 10.1007/s11306-012-0473-x (PMC3651535; doi:10.1007/s11306-012-0473-x)
Supplement: Supplementary file 2 — Supplementary material 2 (PPTX 1066 kb) [file 11306_2012_473_MOESM2_ESM.pptx]

## Slide 1
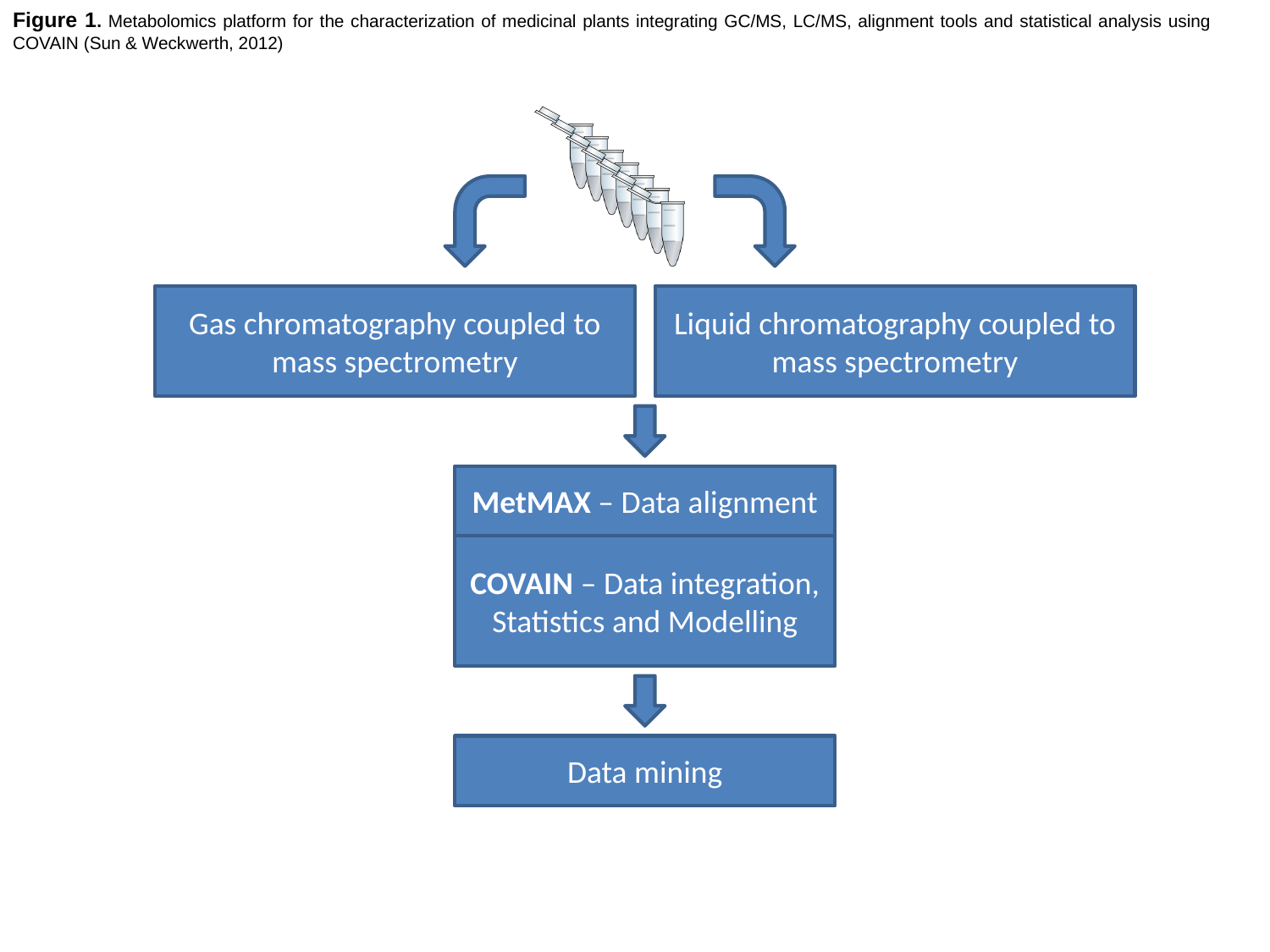

Figure 1. Metabolomics platform for the characterization of medicinal plants integrating GC/MS, LC/MS, alignment tools and statistical analysis using COVAIN (Sun & Weckwerth, 2012)
Gas chromatography coupled to mass spectrometry
Liquid chromatography coupled to mass spectrometry
MetMAX – Data alignment
COVAIN – Data integration, Statistics and Modelling
Data mining

## Slide 2
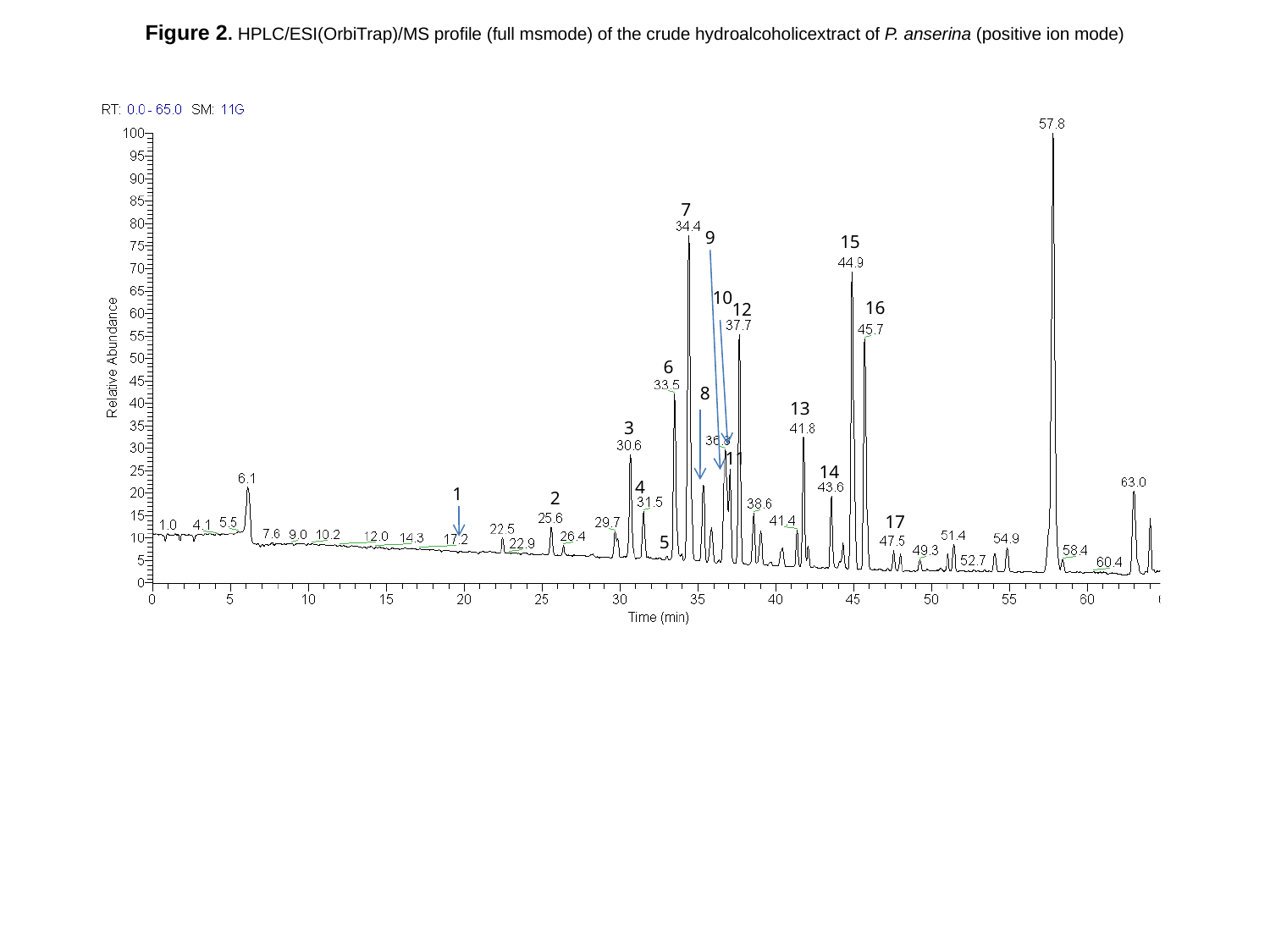

Figure 2. HPLC/ESI(OrbiTrap)/MS profile (full msmode) of the crude hydroalcoholicextract of P. anserina (positive ion mode)
7
9
15
10
16
12
6
13
3
11
14
4
1
2
17
5
8

## Slide 3
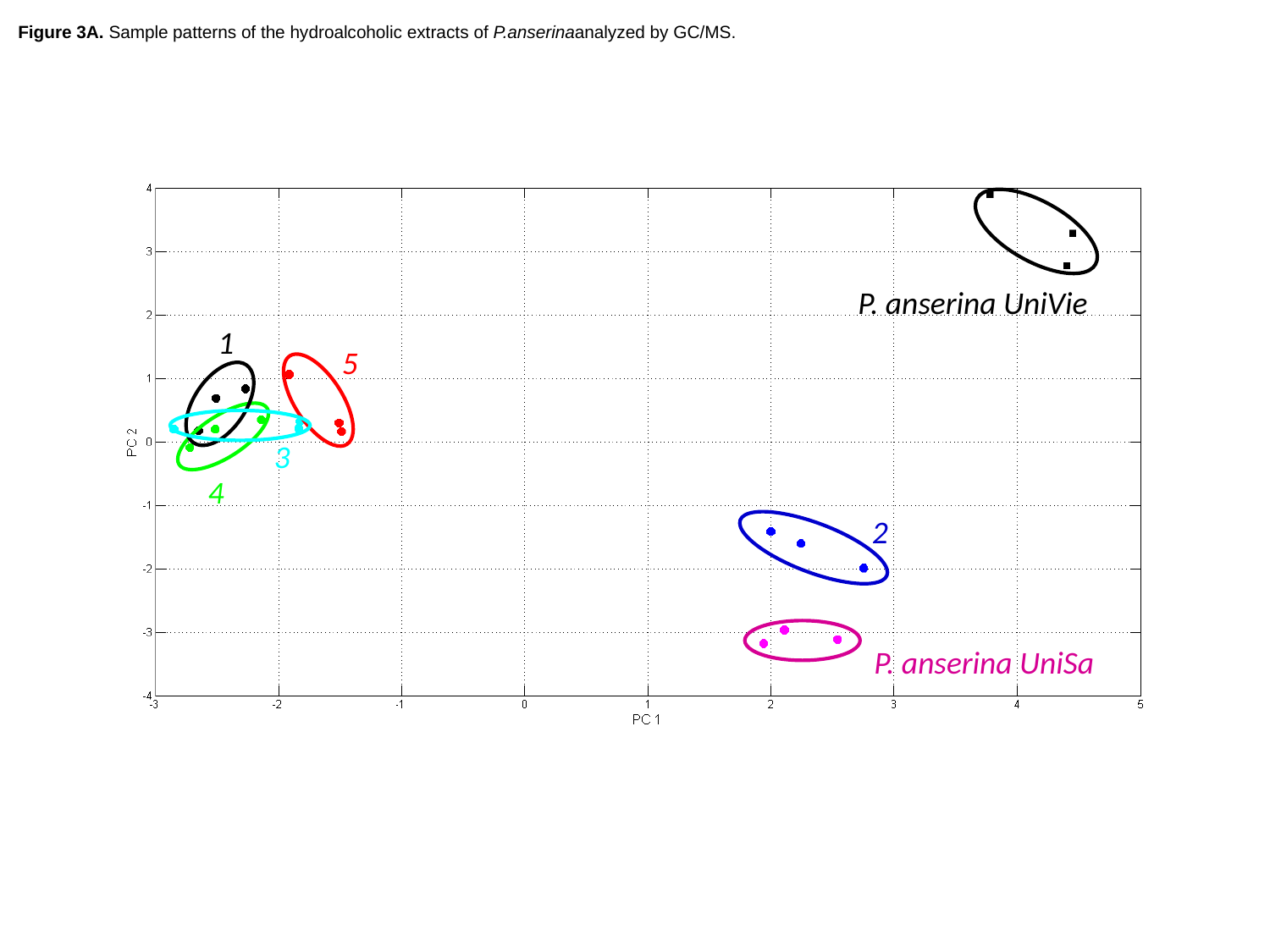

Figure 3A. Sample patterns of the hydroalcoholic extracts of P.anserinaanalyzed by GC/MS.
3
P. anserina UniVie
1
5
4
2
P. anserina UniSa

## Slide 4
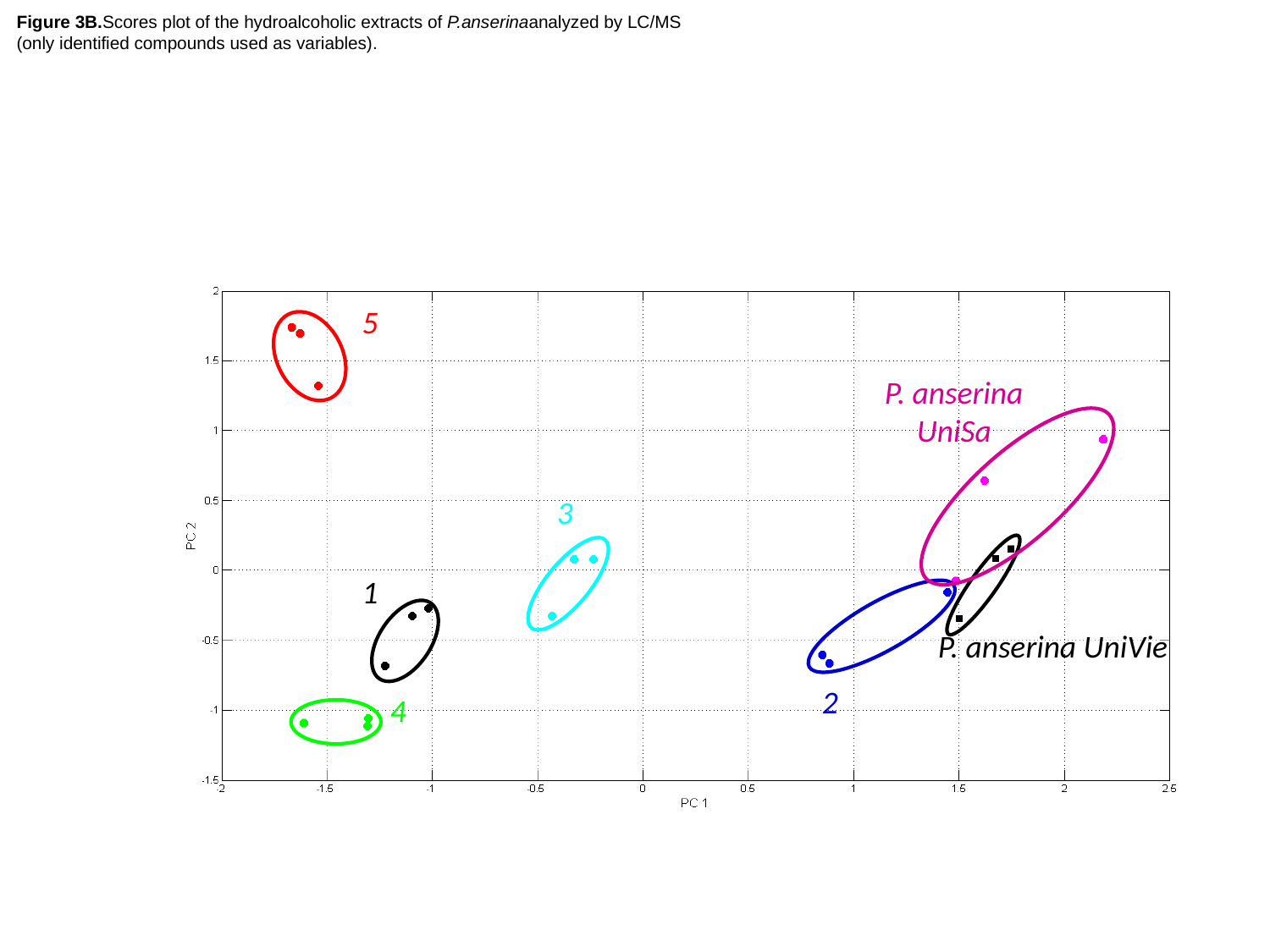

Figure 3B.Scores plot of the hydroalcoholic extracts of P.anserinaanalyzed by LC/MS
(only identified compounds used as variables).
5
P. anserina UniSa
3
1
P. anserina UniVie
2
4

## Slide 5
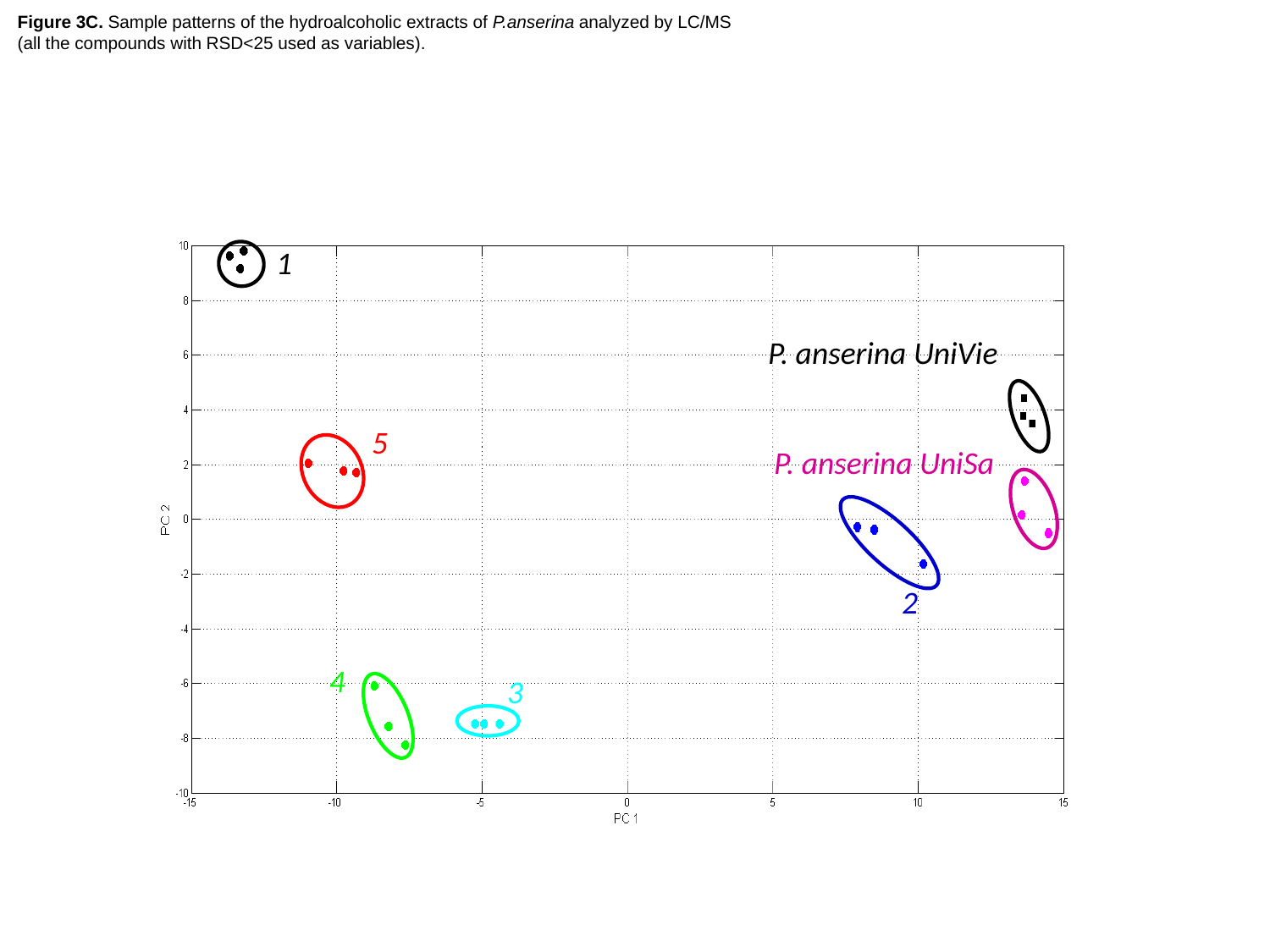

Figure 3C. Sample patterns of the hydroalcoholic extracts of P.anserina analyzed by LC/MS
(all the compounds with RSD<25 used as variables).
1
4
P. anserina UniVie
5
P. anserina UniSa
2
3

## Slide 6
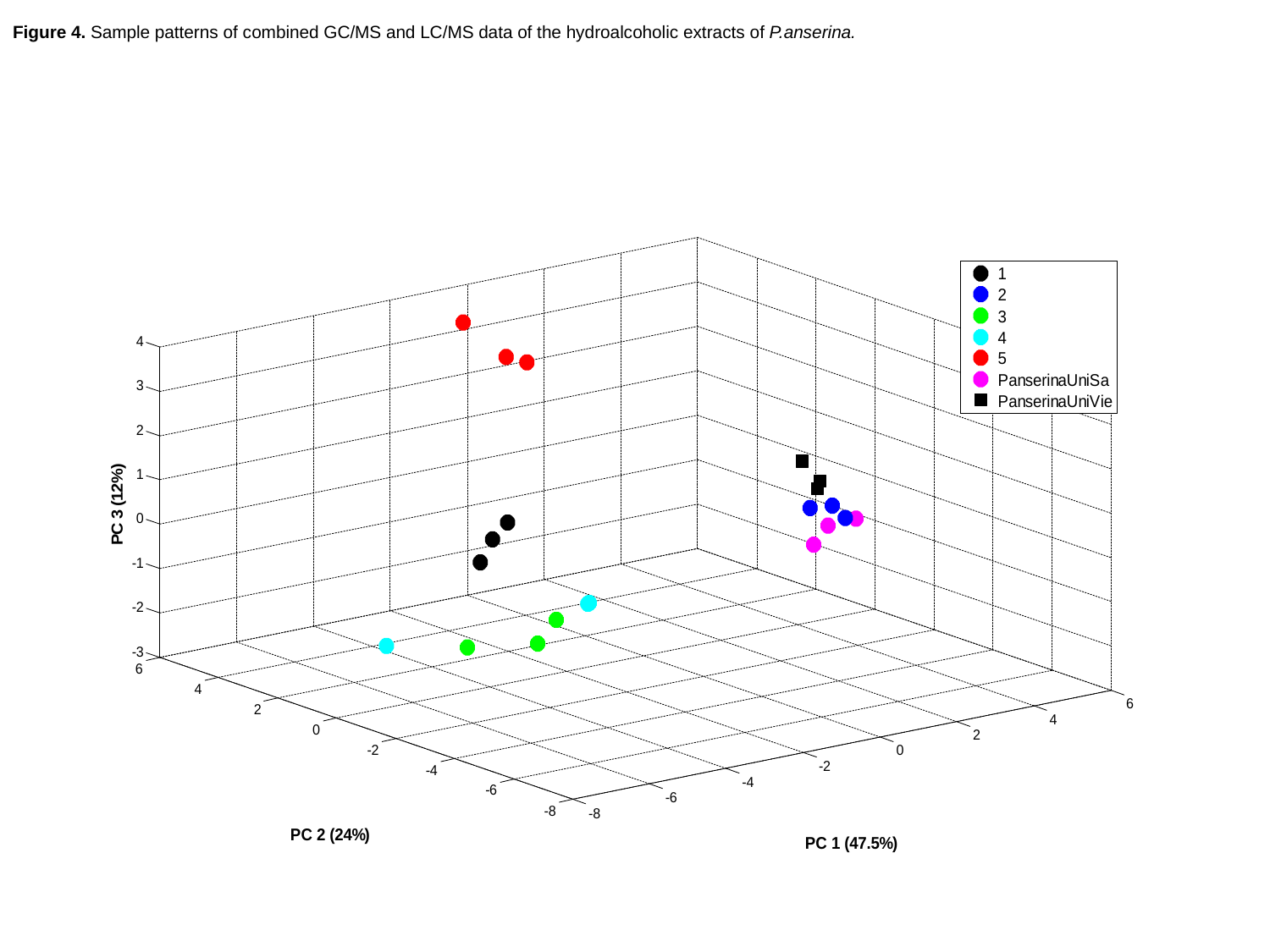

Figure 4. Sample patterns of combined GC/MS and LC/MS data of the hydroalcoholic extracts of P.anserina.

## Slide 7
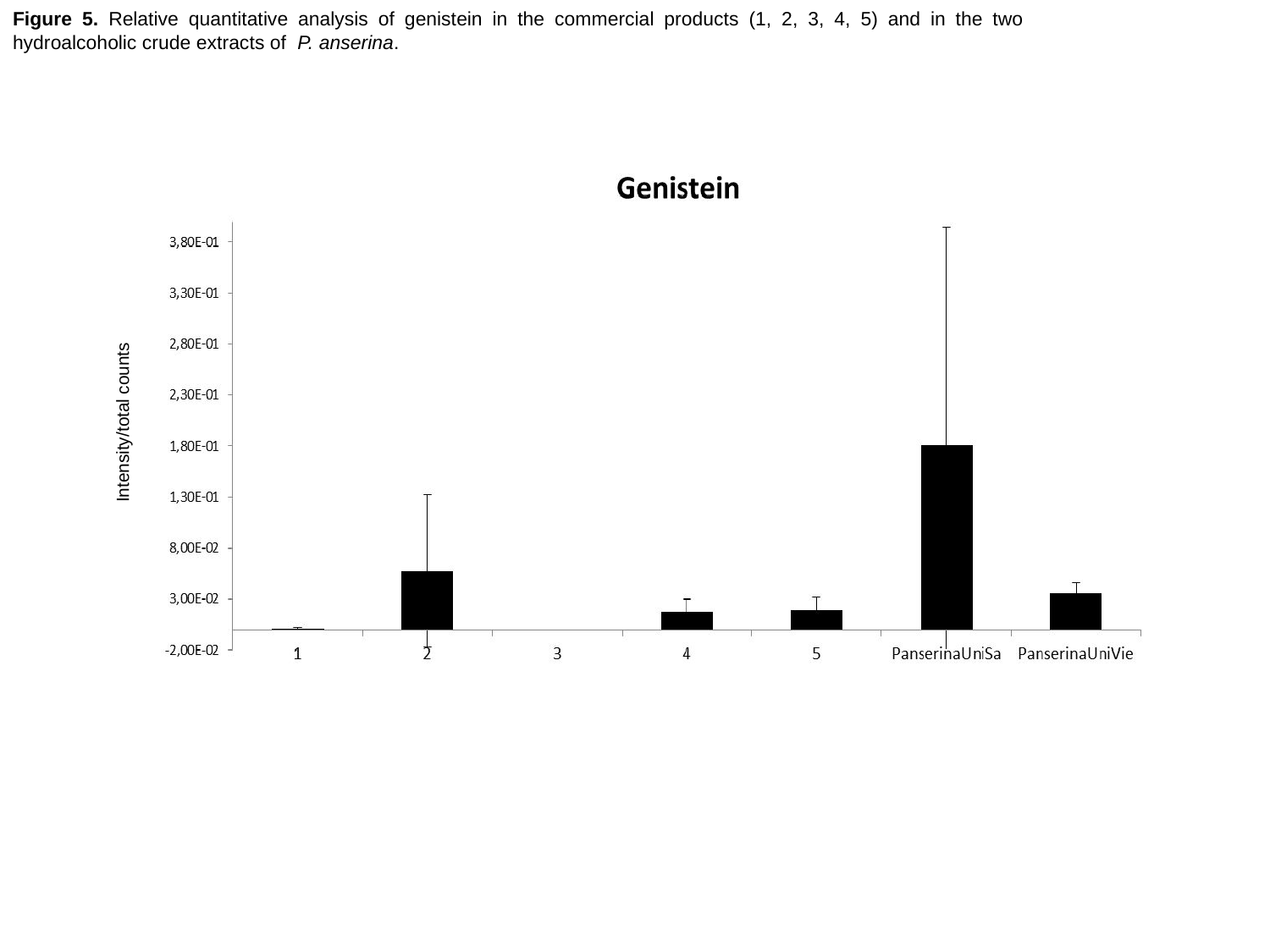

Figure 5. Relative quantitative analysis of genistein in the commercial products (1, 2, 3, 4, 5) and in the two hydroalcoholic crude extracts of P. anserina.
Intensity/total counts

## Slide 8
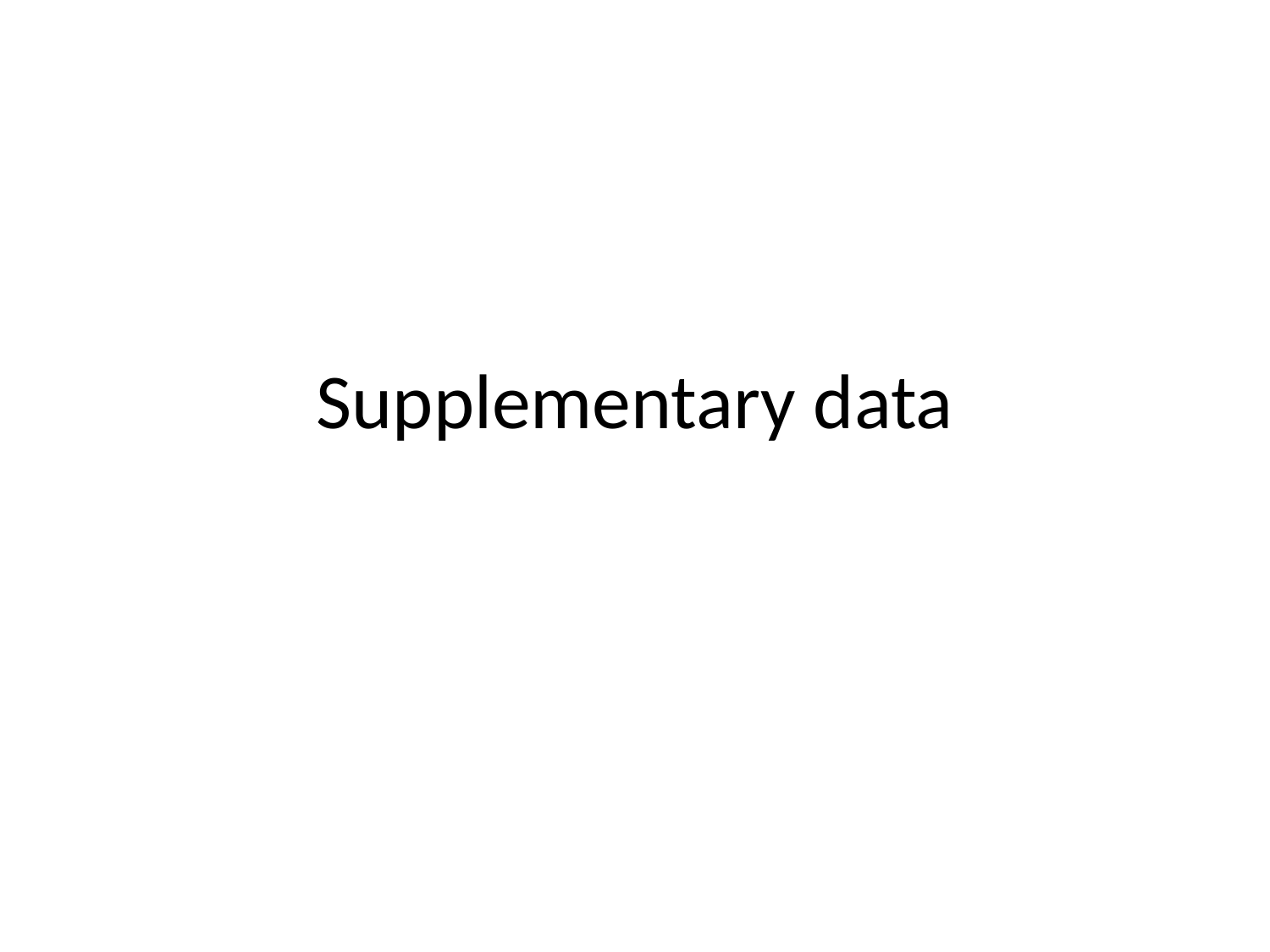

# Supplementary data

## Slide 9
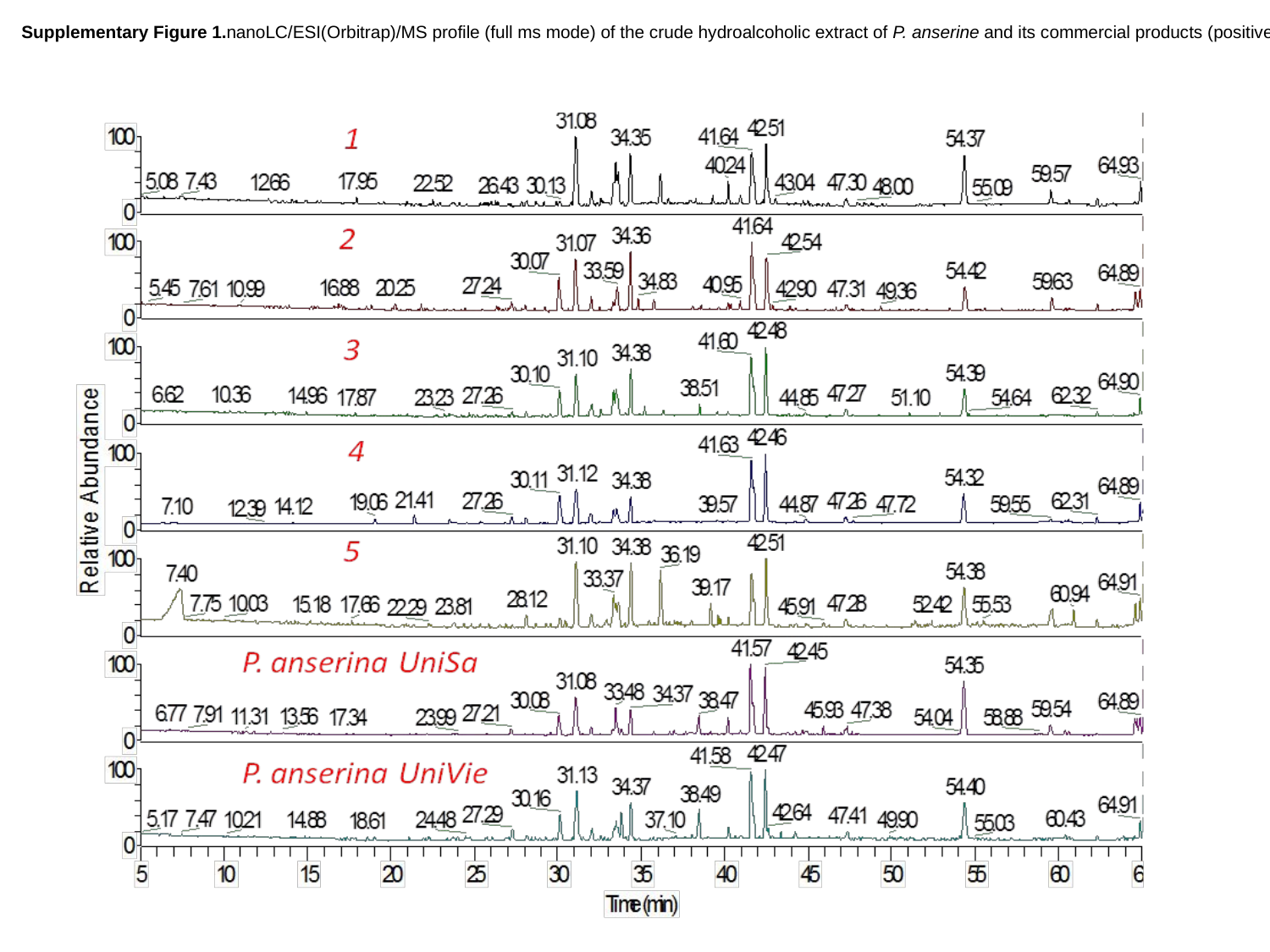

Supplementary Figure 1.nanoLC/ESI(Orbitrap)/MS profile (full ms mode) of the crude hydroalcoholic extract of P. anserine and its commercial products (positive ion mode)

## Slide 10
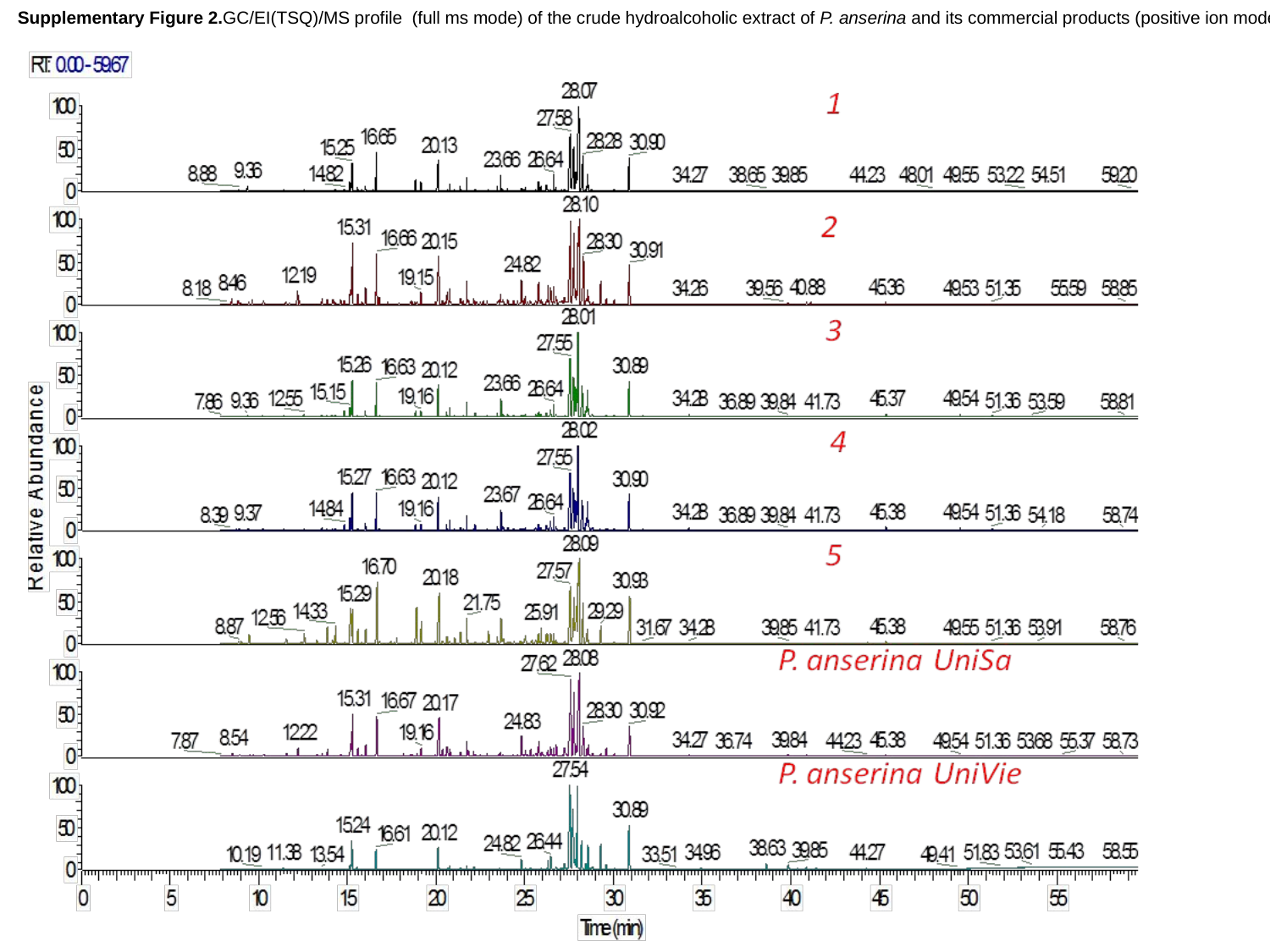

Supplementary Figure 2.GC/EI(TSQ)/MS profile (full ms mode) of the crude hydroalcoholic extract of P. anserina and its commercial products (positive ion mode)

## Slide 11
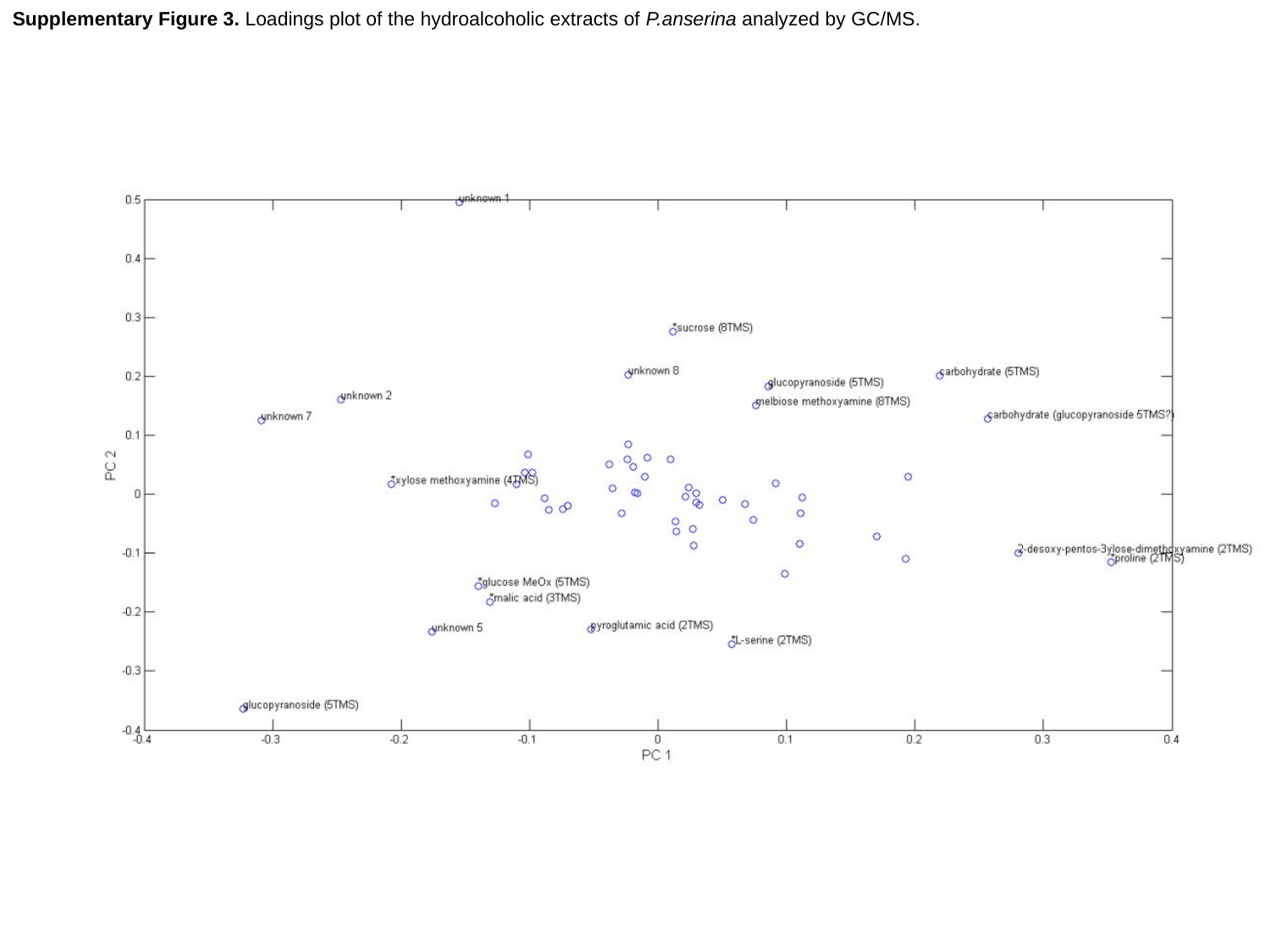

Supplementary Figure 3. Loadings plot of the hydroalcoholic extracts of P.anserina analyzed by GC/MS.

## Slide 12
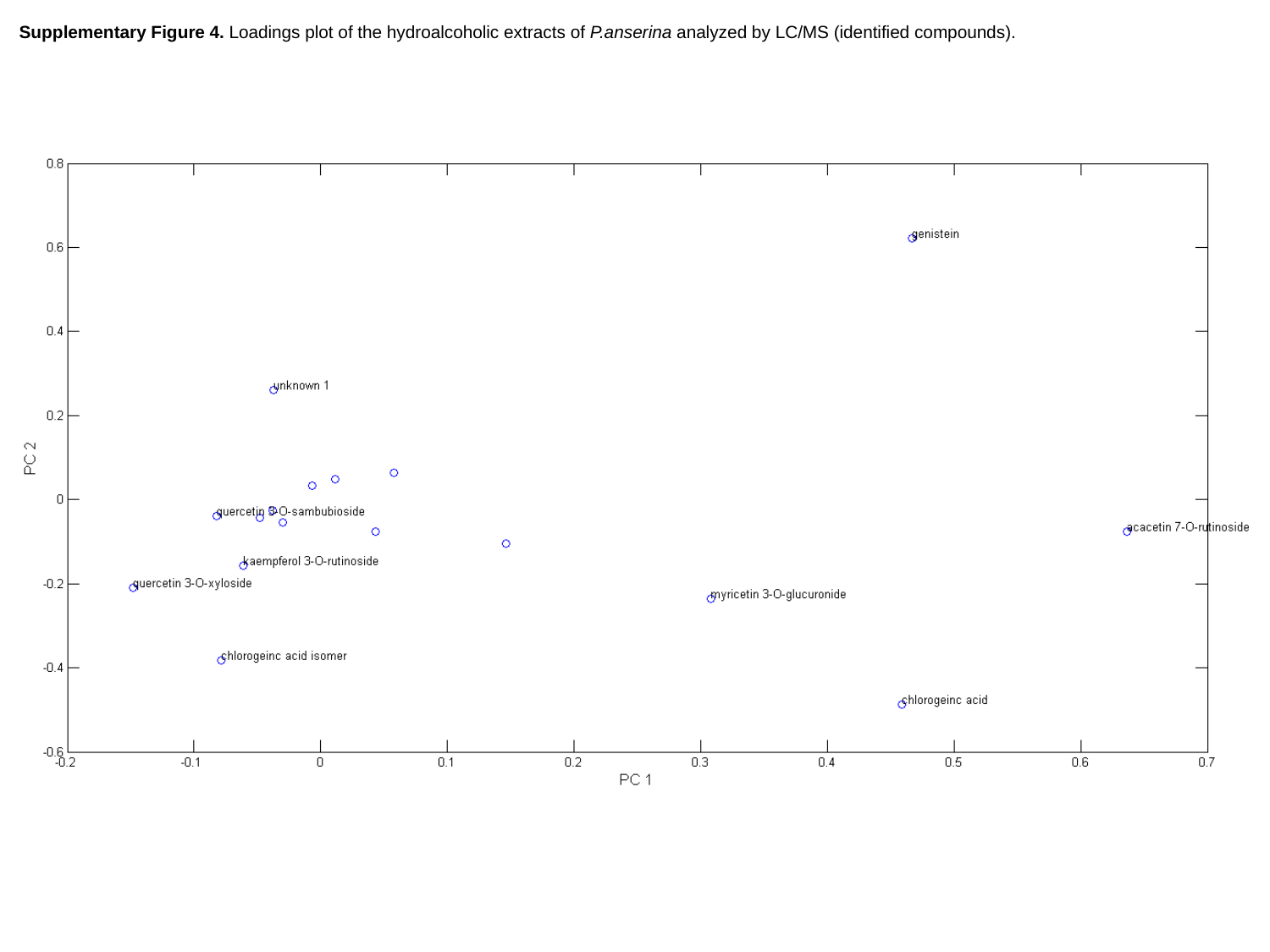

Supplementary Figure 4. Loadings plot of the hydroalcoholic extracts of P.anserina analyzed by LC/MS (identified compounds).

## Slide 13
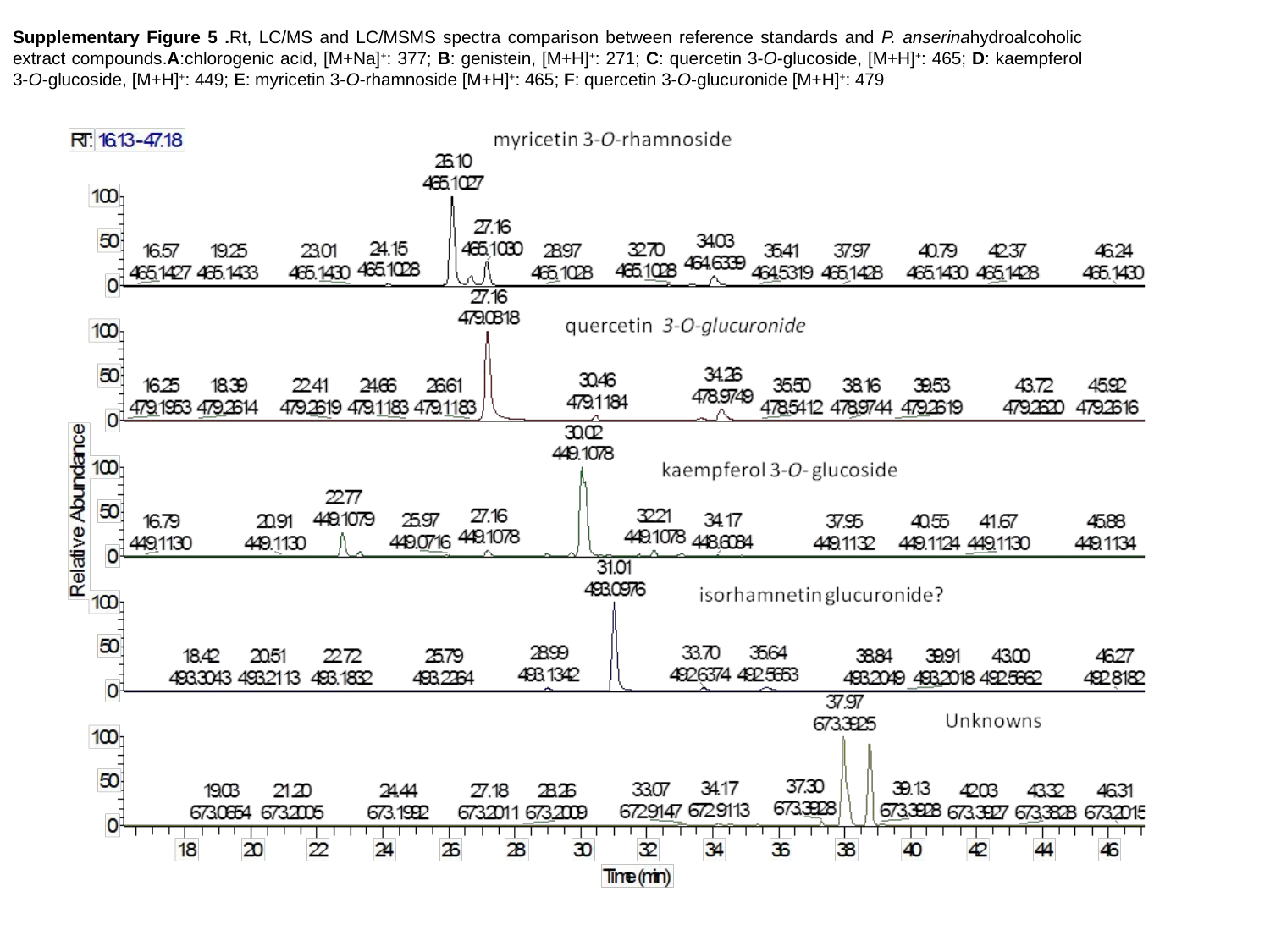

Supplementary Figure 5 .Rt, LC/MS and LC/MSMS spectra comparison between reference standards and P. anserinahydroalcoholic extract compounds.A:chlorogenic acid, [M+Na]+: 377; B: genistein, [M+H]+: 271; C: quercetin 3-O-glucoside, [M+H]+: 465; D: kaempferol 3-O-glucoside, [M+H]+: 449; E: myricetin 3-O-rhamnoside [M+H]+: 465; F: quercetin 3-O-glucuronide [M+H]+: 479

## Slide 14
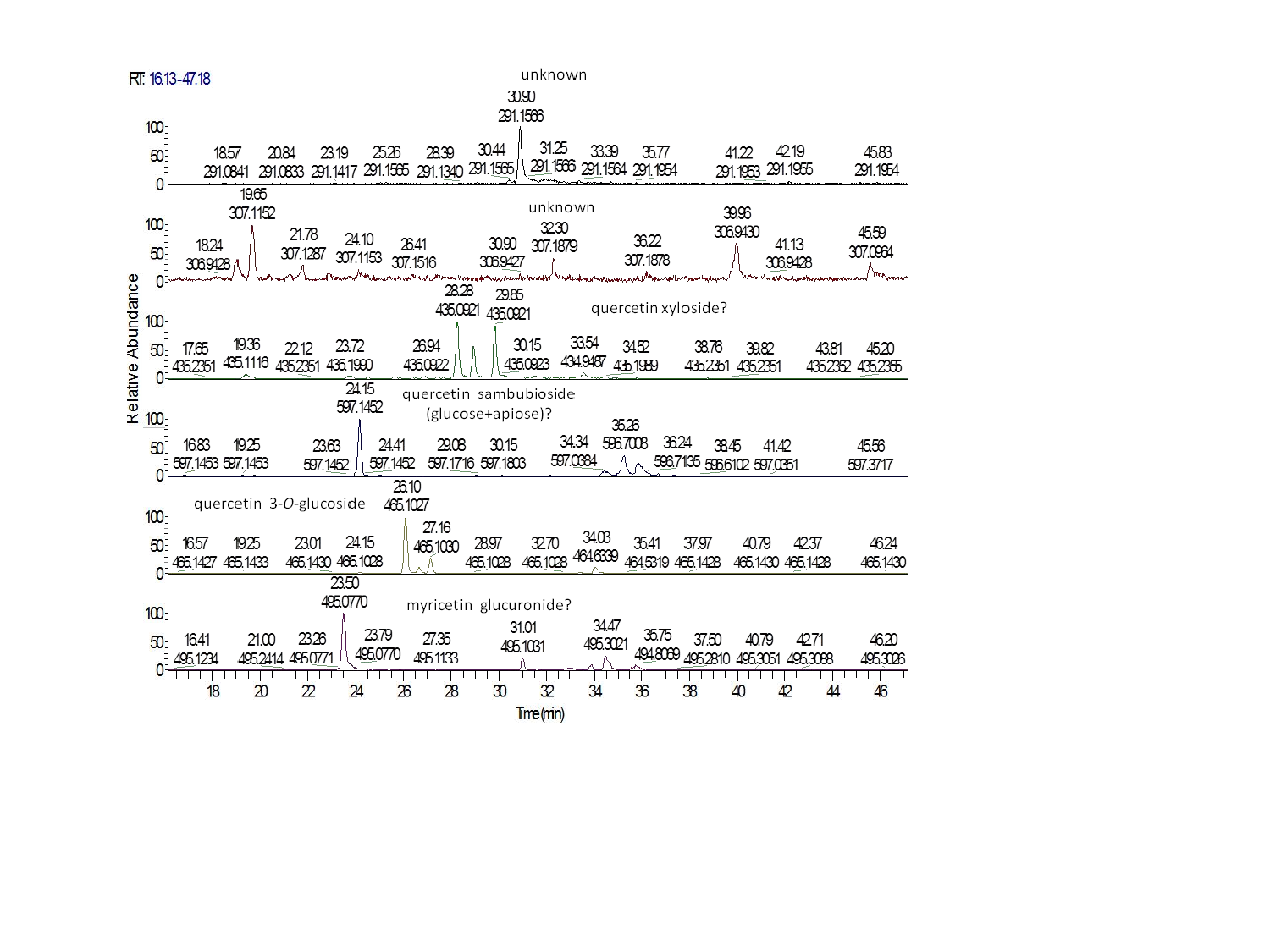

## Slide 15
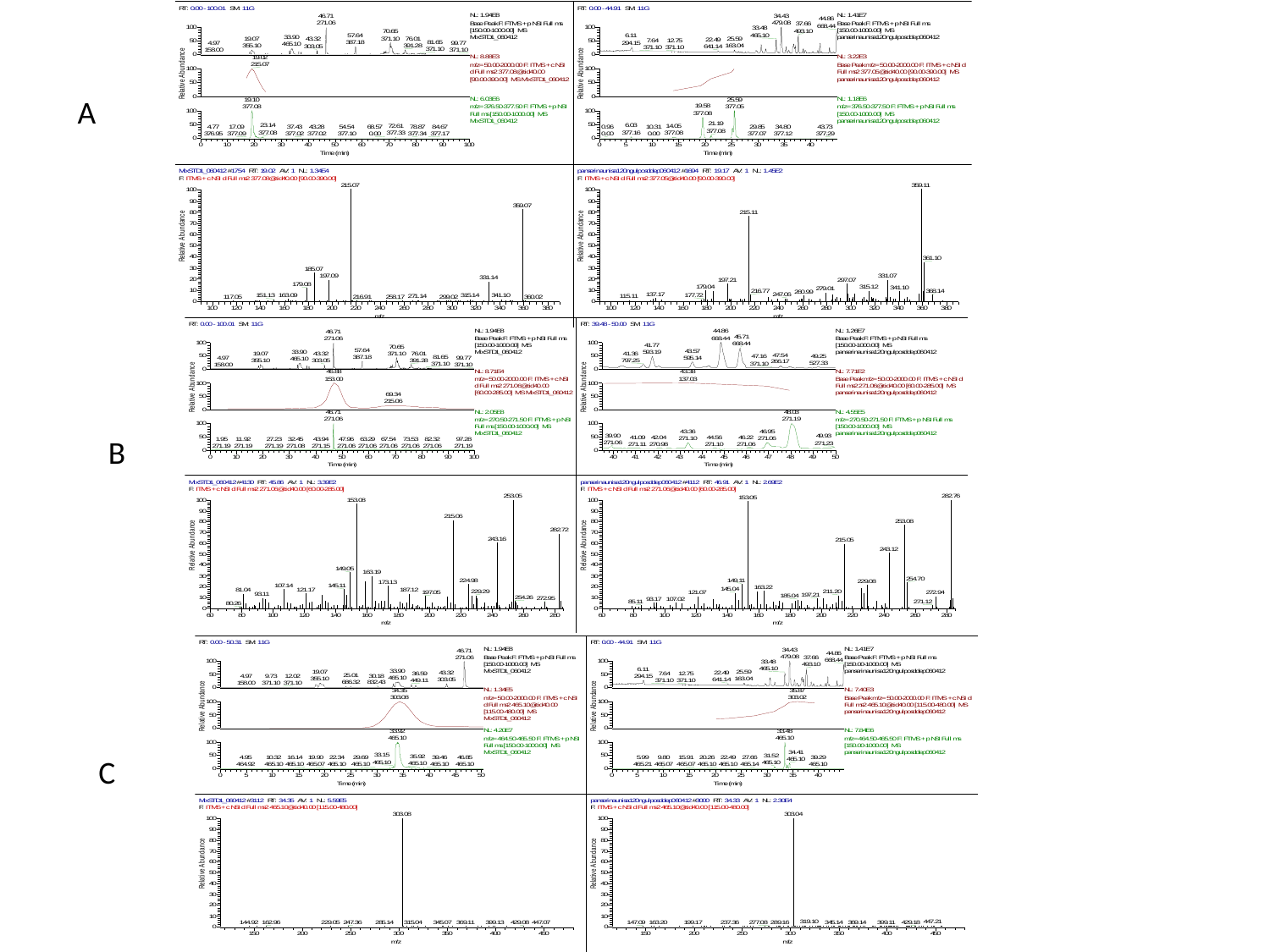

A
B
C

## Slide 16
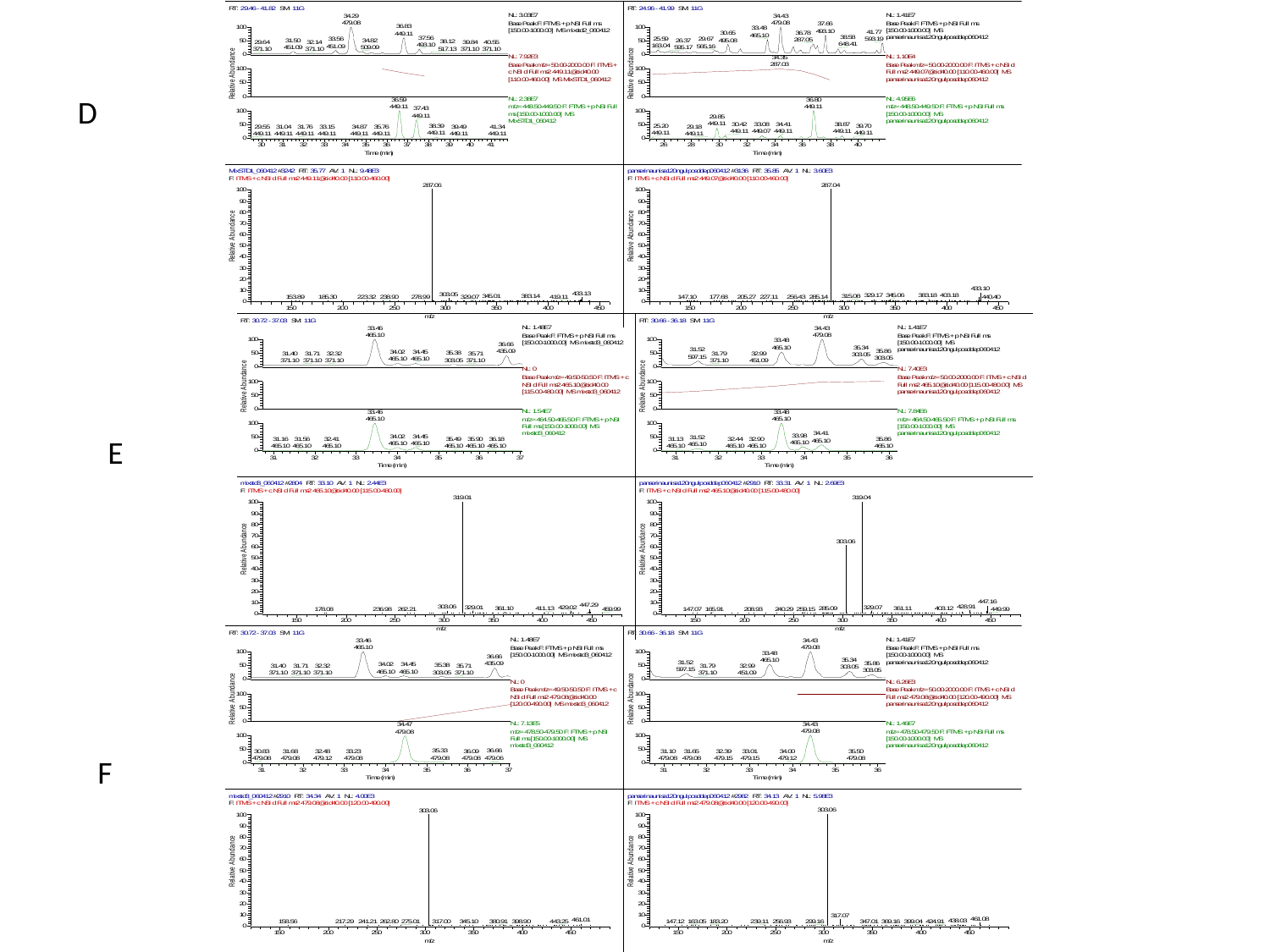

D
E
F
